# Traditional Chinese exercise for non-valvular atrial fibrillation: A protocol for systematic review and meta-analysis

**DOI:** 10.1097/MD.0000000000031829

**Published:** 2022-12-09

**Authors:** Te Wang, Hongguang Jin, Yiqiang Wang, Meixi Liu, Huan Liu, Xing Zhu, Wenping Guo, Chunhui Fan, Yongsheng Huang, Lihong Jiang

**Affiliations:** a Changchun University of Chinese Medicine, Changchun City, Jilin Province, China; b Affiliated Hospital of Changchun University of Chinese Medicine, Changchun City, Jilin Province, China; c The Affiliated TCM Hospital of Guangzhou Medical University, Guangzhou City, Guangdong Province, China.

**Keywords:** efficacy and safety, meta-analysis, non-valvular atrial fibrillation, protocol, traditional Chinese exercise

## Abstract

Traditional Chinese exercises have become an important part of cardiac rehabilitation. It can coordinate the essence, qi, and spirit of the human body, and has the functions of promoting joints, stretching muscles and bones, ventilating and blood circulation, so as to achieve the balance between hardness and softness, and between yin and yang. We hope that the research results based on systematic review and meta-analysis will provide a theoretical basis for the treatment of atrial fibrillation (AF) with traditional Chinese exercise.

**Methods:**

The systematic review will be performed according to the Cochrane Handbook for Systematic Reviews of Interventions. The protocol is being reported in accordance with the Preferred Reporting Items for Systematic Review and Meta-Analysis Protocols Statement. An literature search strategy will be developed and adapted for 9 databases. Searches will be run from the database inception until the date of the search implementation and be updated before the review is completed. Meta analysis will be performed using Review Manager 5.3 and R packages.

**Conclusion:**

This protocol introduces a systematic review and meta-analysis of traditional Chinese exercises in the treatment of nonvalvular AF and will clarify the efficacy and safety of traditional Chinese exercises in the treatment of AF. This will further provide theoretical support for clinical treatment of AF.

## 1. Introduction

Atrial fibrillation (AF) is a common, complex, and age-related tachyarrhythmia. It is mainly caused by many small reentrant loops and atrial rhythm disorder. It is characterized by atrial uncoordinated activity and atrial function deterioration, and is not only seen in almost all organic heart diseases. Nonorganic heart disease can also occur.^[1]^ According to epidemiological data, there were 3.046 million new cases of AF worldwide in 2017, and the prevalence of AF in adults alone is as high as 2%-4%, and it is increasing year by year.^[2]^

AF not only has a high incidence, but also has many serious hazards. Such as increased risk of ischemic stroke and systemic arterial embolis. The risk of ischemic stroke in patients with AF is 4 to 5 times higher than that in patients without AF, which can lead to a mortality rate of nearly 20% and a disability rate of 60%. At the same time, it also increases the risk of heart failure and myocardial infarction, leading to cognitive decline, renal function damage, etc.^[3]^

At present, the treatment of AF includes the restoration and maintenance of sinus rhythm, control of ventricular rate, prevention of thromboembolism and stroke, surgical treatment, and upstream treatment of AF, that is, the management of AF risk factors.^[4]^ Exercise rehabilitation is an important means of rehabilitation for patients with heart disease. Proper and regular physical exercise can improve the exercise ability of patients with AF, which is very beneficial to improve the quality of life.^[5]^ Exercise training is effective in preventing and treating most AF related diseases, reducing cardiovascular risk factors related to AF, thereby reducing cardiac load, risk of new AF, and risk of recurrence after ablation.^[6]^

Traditional Chinese exercise are an important part of heart rehabilitation, including Taijiquan, Baduanjin, Wuqinxi, Yijinjing, and Liuzi Jue. Traditional Chinese exercises harmonize the essence, qi and spirit of the patient by adjusting the body, breath and heart, and play the role of combining hardness with softness and complementing yin and yang.^[7]^ Some scholars used Shensong Yangxin Capsule and Baduanjin Exercise to intervene patients with AF after radiofrequency catheter ablation, and the results showed that it could effectively improve patients’ cardiac function and prevent recurrence of AF after surgery.^[8]^ However, as far as we know, there is no systematic review on the treatment of AF with Traditional Chinese exercise. We aim to conduct a comprehensive systematic and meta-analysis to evaluate the efficacy and safety of Traditional Chinese exercise for AF.

## 2. Methods and analysis

### 2.1. Registration

This protocol will compliant with the Preferred Reporting Items for Systematic Reviews and Meta-Analysis Protocol (PRISMA-P)^[9]^(see S1 Table1, http://links.lww.com/MD/H980, Supplemental Digital Content, which illustrates the PRISMA-P 2015 checklist).The review has been registered in the International Prospective Register of Systematic Reviews (PROSPERO) with the identifier CRD42022366253.

### 2.2. Eligibility criteria

1. Type of study

We will include randomized controlled trials as the primary study design, whether they are blinded or design or not. Studies published in peer-reviewed journals which in English or Chinese will be included in this review.

1. Participant

Adult non-valvular AF participants aged ≥ 18 years, regardless of their primary disease, race, gender, and ethnicity will be included.

1. Intervention

Conventional treatment of western medicine + traditional Chinese exercise (including Taijiquan, Baduan Jin, Yi Jin Jing, Wuqinxi, six characters, etc.). If any one of the treatment group and the control group used other exercise treatment measures, such as other exercise treatment measures, jog, etc., it was excluded.

1. Comparator

Conventional treatment of Western medicine (such as anticoagulation, heart rate control, etc.).

1. Type of outcomes

This review will include studies that focus on the following outcomes, it will be divided into primary outcomes and secondary outcomes: Primary outcomes (such as stroke, systemic embolism); Secondary outcomes (such as diversion rate, recurrence rate, maintenance rate of sinus rhythm, 6-minute walking test, etc.).

### 2.3. Search strategy

To identify studies for inclusion in this review, the following electronic databases will be searched for relevant articles published up to November 2022: PubMed, EMBASE, the Cochrane Library, SinoMed, CNKI, VIP, Wanfang Data, and International Clinical Trials Register (ICTRP) Search Portal and ClinicalTrials.gov. In the month when the article is to be completed, the literature will be reviewed again to observe whether there are new relevant literatures. Screening reference lists and consulting experts will be performed in this time frame. Studies in accordance with the PICOS will be considered. Key search terms (MeSH and Free words) used for our searches are “atrial fibrillation” or “Tai Ji” or “Baduanjin” and “RCTs,” etc.

### 2.4. Study selection

**Figure 1.**
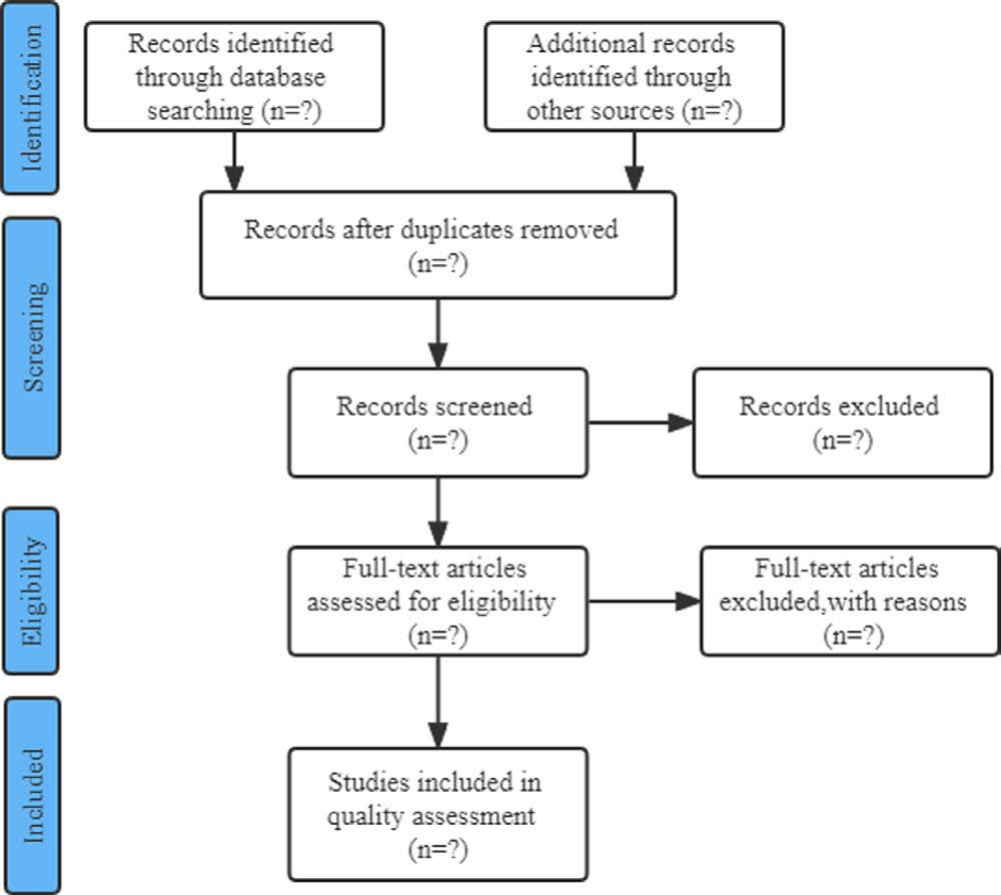
Flow diagram for study.

Endnote X9.1 software will be used to collect citations and removed the duplicated records Two authors (WT and LMX) will independently screen titles and abstracts of deduplicated articles and remove those that do not fit the eligibility criteria;^[2]^ Then recheck the full text of the remaining articles, the eligible literature was included, and the unqualified literature was excluded. A third reviewer (JHG) will be consulted, in case of disagreement. Excluded literature and its reasons for exclusion will be recorded. The selecting process will follow the PRISMA flow diagram^[9]^, see Figure 1.

### 2.5. Data extraction

Two authors will extract the data independently, and the collation of the extracted content will be done using Excel spreadsheet 2019. The two authors will independently perform data extraction on the following informations: including study characteristics (such as published title, author name, journal name, the country where the study was conducted, year of publication, etc,), participants (such as male-female ratio, average age, disease stage, etc.), interventions: (such as Tai Ji, Baduanjin, Yi jin jing, etc.), outcomes: (including primary outcomes: stroke, systemic embolism; secondary outcomes: diversion rate, recurrence rate, maintenance rate of sinus rhythm, 6-minute walking test, etc.).If the cross-examination results are inconsistent, could consultation with a third author (JHG). If necessary, we will contact the author by phone or email to obtain data.

### 2.6. Assessing risk of bias

Risk of bias will assess independently according to the Cochrane risk of bias tool by two researchers (WT and LMX) for included articles.^[10]^ The following entries will be assessed: sequence generation, allocation concealment, blinding of participants and personnel, blinding of outcome assessment, incomplete outcome data, selective reporting, and other bias(baseline imbalance between groups of participants, differential diagnostic activity, etc.). The assessment will be graphed and use Review Manager 5.3 software.

### 2.7. Method for data synthesis

1. Meta-analysis

We will extract the levels of outcome measures before and after treatment and during follow-up arm of included studies to calculate an individual study relative risk RR/OR/HR and 95% confidence interval. Effect estimates were summarized using fixed random-effects meta-analysis and expressed as RRs/ORs/HRs and 95% confidence intervals. If the study showed large heterogeneity, a random-effects model was used to synthesis the data. Review Manager 5.3 software^[11]^ will be used to conduct meta analyses.

1. Heterogeneity assessment

We will estimate heterogeneity using the Cochrane *Q* test^[12]^ and assessed the extent of heterogeneity with *I*², which expresses the proportion of between-group variability that is attributable to heterogeneity rather than chance. *I*² 75% typically is held to signify high heterogeneity, whereas 25% and 50% correspond to low and medium levels of heterogeneity, respectively.

1. Publication bias

If the number of studies exceeds 10, we will verify the publication offset using the funnel plot and the Begg test. If there is a publication offset, we will use the trim and fill method to evaluate the impact of the offset on the results of the article.

1. Sensitivity analysis

Sensitive analysis will be used to verify the robustness of the results. According to leave-one-out strategy^[13,14]^, validation can be performed.

1. Subgroups analysis

Subgroup analyses will conduct according to different sample size, treatment course, and follow-up period etc. to explore the potential sources of heterogeneity in the treatment outcomes. We will explain the sources of heterogeneity based on the results of the subgroup analysis.

### 2.8. Quality of the evidence

The certainty of evidence will be graded for each outcome, from a rating of HIGH to VERY LOW by following the Grading of Recommendations Assessment, Development, and Evaluation (GRADE) approach.^[15]^ Including risk of bias, imprecision, inconsistency, indirectness, and publication bias will be assessed.

### 2.9. Ethics and dissemination

As the data we used were secondary data, ethical approval or informed consent was not required for this systematic review. The findings of this review will be disseminated through peer-reviewed publications and conference reports.

### 2.10. Patient and public involvement

No patients and/or the public are involved in the study.

## 3. Discussion

Nonvalvular atrial fibrillation refers to AF without rheumatic mitral stenosis and mechanical or biological artificial heart valves or mitral valve repair.^[16]^ It is a common type of AF. At present, the clinical treatment of AF is usually a combination of drugs and nondrugs. But at the same time, patients are also faced with the problems of anticoagulant intolerance, adverse effects of antiarrhythmic drugs on external organs of the heart, high costs of radiofrequency ablation and left atrial appendage occlusion, and postoperative recurrence.^[17]^ Traditional Chinese exercises have the functions of facilitating joints, stretching muscles and bones, and unblocking qi and blood. The most outstanding advantage of traditional Chinese exercises is safe and no side effects. Regular exercise can achieve the goal of curing diseases and keeping healthy. In recent years, the application of traditional exercises in cardiac rehabilitation has gradually increased. With the aging of the population, the number of elderly patients with AF gradually increases. The gentle and slow traditional Chinese exercises are just suitable for the elderly. Therefore, we hope that the research results based on systematic review and meta-analysis will provide a theoretical basis for the treatment of AF with traditional Chinese exercise.

## Author contributions

Conceptualization: Te Wang.

Data curation: Hongguang Jin.

Formal analysis: Meixi Liu and Xing Zhu.

Funding acquisition: Yiqiang Wang and Lihong Jiang.

Investigation: Yongsheng Huang.

Methodology: Te Wang and Chunhui Fan.

Supervision: Yongsheng Huang.

Validation: Huan Liu and Wenping Guo.

Visualization: Hongguang Jin.

Writing—original draft: Te Wang.

Writing—review and editing: Yongsheng Huang, Lihong Jiang and Hongguang Jin.

## Supplementary Material


